# Ventriculitis incidence and outcomes in patients with aneurysmal subarachnoid hemorrhage: a prospective observational study

**DOI:** 10.62675/2965-2774.20250076

**Published:** 2025-01-15

**Authors:** Ricardo Turon, Pedro Kurtz, Carla Rynkowski, Letícia Petterson, Bruno Gonçalves, Vanessa de Caro, Marco Prazeres, Fernando Augusto Bozza, Cassia Righy

**Affiliations:** 1 Department of Neurointensive Care Instituto Estadual do Cérebro Paulo Niemeyer Rio de Janeiro RJ Brazil Department of Neurointensive Care, Instituto Estadual do Cérebro Paulo Niemeyer - Rio de Janeiro (RJ), Brazil.; 2 Department of Intensive Care Medicine Hospital Cristo Redentor Porto Alegre RS Brazil Department of Intensive Care Medicine, Hospital Cristo Redentor - Porto Alegre (RS), Brazil.; 3 Instituto D’Or de Pesquisa e Ensino Rio de Janeiro RJ Brazil Instituto D’Or de Pesquisa e Ensino - Rio de Janeiro (RJ), Brazil.

**Keywords:** Subarachnoid hemorrhage, Cerebral ventriculitis, Ventriculostomy, Cross infection, Drainage

## Abstract

**Objective:**

To define the incidence of ventriculostomy-associated infections and their impact on the mortality and functional outcomes of patients with aneurysmal subarachnoid hemorrhage.

**Methods:**

We prospectively included all consecutive adult aneurysmal subarachnoid hemorrhage patients admitted to the neurological intensive care units of the *Instituto Estadual do Cérebro Paulo Niemeyer* (Rio de Janeiro, Brazil) and *Hospital Cristo Redentor* (Rio Grande do Sul, Brazil) who required external ventricular drains from July 2015 to December 2020. Daily clinical and laboratory variables were collected at admission and during the hospital stay. The presence of ventriculostomy-associated infections was evaluated daily, according to the Centers for Disease Control and Prevention and Infectious Diseases Society of America criteria. Hospital and 12-month outcomes were compared between patients with and without ventriculostomy-associated infections via both univariate and multivariate analyses.

**Results:**

Out of the 676 patients screened, 271 received external ventricular drains (40%) and were included in the study. The mean age was 54 years (IQR 46–63), 198 were female (72%), 47% had poor grade status (World Federation of Neurological Surgeons scale 4 and 5), and 75% had modified Fisher 3 or 4. The mean time from admission to external ventricular drain placement was 8.8 days. Ventriculostomy-associated infections developed in 127 patients (47%), and the mean time from external ventricular drain to ventriculostomy-associated infection diagnosis was 4.4 days. Hospital and 12-month mortality rates did not differ between the ventriculostomy-associated infection group and the nonventriculostomy-associated infection group (36% *versus* 40% and 43% *versus* 49%, respectively). Poor functional outcomes, defined as modified Rankin scores of 4 to 6, showed no difference between groups at hospital discharge (ventriculostomy-associated infections 75% *versus* nonventriculostomy-associated infections 73%; p = NS) or at 12 months (ventriculostomy-associated infections 49% *versus* nonventriculostomy-associated infections 53%; p = NS).

**Conclusion:**

Ventriculostomy-associated infections are common complications after aneurysmal subarachnoid hemorrhage. Although it was not associated with hospital mortality or functional outcomes in our cohort, improving diagnostic accuracy and preventive measures is essential for better understanding the long-term impact of one of the most severe infectious complications after aneurysmal subarachnoid hemorrhage.

## INTRODUCTION

Aneurysmal subarachnoid hemorrhage (SAH) is an acute cerebrovascular disease with devastating consequences, including high mortality and long-term functional impairment among survivors.^1^ Infectious complications affect approximately 30% of patients, among which ventriculostomy-associated infections (VAIs) can be severe and potentially fatal.^2,3^ The incidence of VAIs depends on diagnostic criteria, severity profiles, and health structures, with large variations observed among high-, low-, and middle-income countries.^4-8^ Data on the impact of the VAI on SAH mortality and functional outcomes are conflicting. Moreover, to the best of our knowledge, no study in South America has investigated the incidence of VAI or its impact on outcomes, which could influence local health policies and management guidelines. This study aimed to determine the incidence of VAI in patients with SAH in a middle-income country and the associations of this infectious complication with mortality and functional outcomes.

## METHODS

### Design and setting

We prospectively included all consecutive adult SAH patients admitted to the neurological intensive care unit (ICU) of the *Instituto Estadual do Cérebro Paulo Niemeyer* (Rio de Janeiro, Brazil) and *Hospital Cristo Redentor* (Rio Grande do Sul, Brazil) from May 2015 to December 2020; these patients had an aneurysmal etiology and required an external ventricular drain (EVD). The aforementioned hospitals are reference centers for neurovascular diseases, admitting approximately 70 - 100 SAH patients per year from a state-wide public health care system in Brazil.

The present study was approved by the Ethics Review Board of the *Instituto Nacional de Infectologia Evandro Chagas* - *Fundação Oswaldo Cruz* (CAAE 52532815.4.0000.5262) and the *Hospital Cristo Redentor* (CAAE 27362719.6.0000.5530). The data were deidentified by assigning each patient a unique study number. Aneurysmal subarachnoid hemorrhage was diagnosed by findings from the initial computed tomography (CT) scan or by xanthochromia in the cerebrospinal fluid if the CT scan was normal. We excluded patients who were admitted after 30 days of presumed hemorrhagic ictus, who were pregnant, or who had a life expectancy of less than 48 hours after admission to the ICU.

### Clinical assessment

Demographic data, social and medical history, and clinical features at onset were obtained shortly after admission. Neurological status was assessed with the Glasgow Coma Scale (GCS)^9^ and the World Federation of Neurological Surgeons scale (WFNS).^10^ Admission and follow-up CT scans during hospitalization were categorized by using the modified Fisher scale (mFs)^11^ and for the presence of global cerebral edema and infarction. Daily clinical and laboratory follow-up data were analyzed during the first 14 days of hospitalization or until ICU discharge (if the length of stay was less than 14 days). Data were collected daily by two investigators via an electronic case report form (through Research Electronic Data Capture - REDCap). For all patients, the VAI was evaluated daily, according to the Centers for Disease Control and Prevention (CDC) criteria (Table 1S - Supplementary Material). The clinical variables and the diagnosis of infection were validated by the research study team and through adjudication by an independent infectious disease specialist.

### Outcome assessment

The main outcome was functional status, which was evaluated by the modified Rankin scale (mRS)^12^ at hospital discharge and 12 months after hospital admission. Long-term outcomes were evaluated through telephone interviews by a team of properly trained health care professionals, and the survey form was validated for mRs.^13^ Loss to follow-up was considered for any patient whose data were unavailable after hospital discharge. The functional outcomes were dichotomized into unfavorable (mRs 4 to 6) and favorable outcomes (mRs 0 to 3). Hospital complications after SAH were diagnosed weekly by the clinical team and adjudicated weekly by the research study team. Delayed cerebral ischemia was defined as otherwise unexplained clinical deterioration (such as a new focal deficit, a decrease in the level of consciousness, or both) or a new infarct shown on CT scan that was not visible on admission or immediate postoperative CT scan, or both, after exclusion of other potential causes of clinical deterioration. Postoperative deterioration due to operative complications was defined as any neurological worsening or new infarct within 48 hours of the aneurysm repair procedure. Other complications, such as hydrocephalus (defined as the need for cerebrospinal fluid drainage), re-bleeding, vasospasm (defined as arterial narrowing on cerebral angiogram or a mean velocity higher than 120cm/s and Lindegaard index higher than 3 on transcranial Doppler), and seizures, were also recorded. Aneurysm re-bleeding was defined as acute neurologic deterioration with a new hemorrhage apparent on a CT scan.

### Statistical analysis

Data are presented as the means (standard deviations) or medians (interquartile ranges) for continuous variables and as absolute numbers and percentages for categorical variables. Univariate associations were tested by using the chi-square test or Fisher’s exact test for categorical variables, a two-tailed t-test for normally distributed continuous variables, and the Mann–Whitney U test for nonnormally distributed continuous variables. Multilevel logistic regression was used to evaluate the associations of the VAI with mortality and functional outcomes after adjusting for known predictors and significant covariates. Potential multicollinearity between the parameters of the final regression model was assessed by calculating tolerance and variance inflation factor coefficients. Interaction terms were tested and remained in the final model if they were significant. Significance was set at 0.05 for all analyses. We performed all analyses in R project version 4.0.2 with the lme4 package. All analyses were performed using the statistical software R.

## RESULTS

### Baseline characteristics

**Figure 1:**
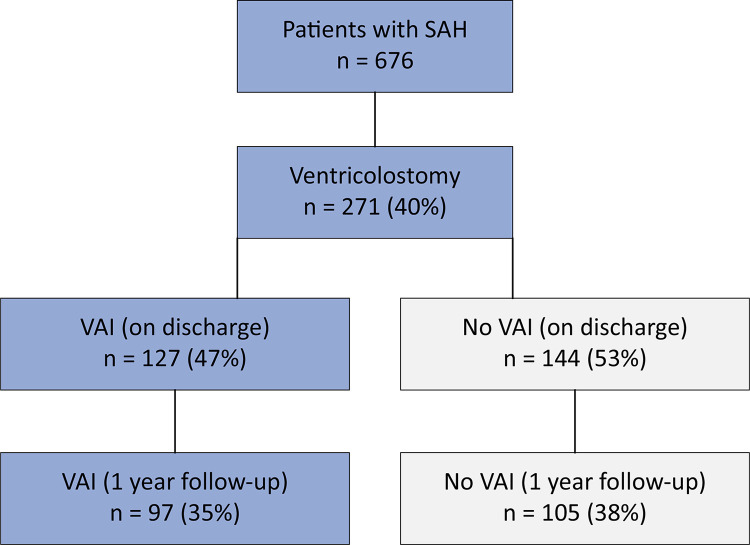
Included patients.

Six hundred and seventy-six patients were screened for study enrollment (Figure 1). Two hundred seventy-one patients needed EVD (40%). There were no patients admitted to the ICU who met the exclusion criteria during the period of the study. Among the EVD patients, the mean age was 54 years (interquartile range [IQR] 46 - 63), 72% were female (n = 198), 47% presented poor baseline grade status (n = 128 with WFNS 4 and 5), and 75% were admitted with significant subarachnoid bleeding (n = 205 presenting with mFs 3 and 4). Patient demographic and baseline characteristics are detailed in table 1.

SAH - Aneurysmal subarachnoid hemorrhage; VAI - ventriculitis-associated infection.

**Table 1:** Demographics and baseline characteristics of patients who required an external ventricular drain

	All patients n = 271	VAI n = 127	Non-VAI n = 144	p value
Age	55 (46 - 63)	54 (45 - 63)	55 (46 - 65)	
Sex female	198 (73)	96 (76)	102 (71)	p > 0.05
Arterial hypertension	174 (64)	87 (68)	87 (60)	p > 0.05
Diabetes mellitus	26 (10)	12 (9)	14 (10)	p > 0.05
Alcoholism	29 (11)	10 (8)	19 (13)	p > 0.05
Smoking	118 (44)	56 (44)	62 (43)	p > 0.05
WFNS grade (I - V) *				
I	71 (26)	39 (31)	32 (22)	
II	54 (20)	27 (21)	27 (19)	p > 0.05
III	16 (6)	6 (5)	9 (6)	p > 0.05
IV	65 (24)	31 (24)	34 (24)	p > 0.05
V	64 (24)	22 (17)	41 (28)	p < 0.05
Poor grade (WFNS IV, V)	129 (48)	54 (43)	75 (52)	p > 0.05
Good grade (I, II, III)	141 (52)	73 (57)	68 (47)	p > 0.05
Modified Fisher (0 - 4) †				
0 and 1	27 (10)	7 (6)	20 (14)	
2	35 (13)	15 (12)	20 (14)	p > 0.05
3	80 (30)	37 (29)	43 (30)	p > 0,05
4	125 (46)	66 (52)	59 (41)	p < 0.05
2, 3 and 4	240 (89)	118 (93)	122 (85)	p < 0.05

VAI - ventriculitis-associated infection; WFNS - World Federation of Neurological Surgeons. * Compared with World Federation of Neurological Surgeons scale I; † Compared with modified Fisher scale 0 or 1. The results are expressed as the means (interquartile ranges) and n (%).

### Incidence of ventriculostomy-associated infection

The time from ictus to EVD placement was 3 (IQR 1 - 7) days, and the time from EVD placement to VAI diagnosis was 6 (IQR 4 - 9) days. In the population with EVD use, 180 (66%) patients underwent mechanical ventilation, and the incidence of VAI during the hospital stay was 47% (n = 127). Poor clinical grade with WFNS V was less common (17% *versus* 28%; p < 0.05), and a CT scan with a modified Fisher classification of 4 was more common (52% *versus* 41%; p < 0.05) in the VAI group than in the non-VAI group. All other baseline characteristics and complications were similar between the groups (Table 2).

**Table 2:** Follow-up and outcomes of patients who required external ventricular drains

	All patients n = 271	VAI n = 127	Non-VAI n = 144	p value
Hydrocephalus	179 (66)	80 (63)	99 (69)	p > 0.05
Mechanical ventilation	180 (66)	81 (64)	99 (69)	p > 0.05
Vasospasm	108 (40)	54 (43)	54 (37)	p > 0.05
Rebleeding	28 (10)	13 (10)	15 (10)	p > 0.05
Postoperative neurologic deterioration	73 (27)	35 (28)	38 (26)	p > 0.05
Delayed cerebral ischemia	95 (35)	43 (34)	52 (36)	p > 0.05
Hospital mortality	104 (38)	46 (36)	58 (40)	p > 0.05
12-month mortality	124 (46)	54 (43)	70 (49)	p > 0.05
Poor outcome at discharge (modified Rankin 4 - 6)	200 (74)	95 (75)	105 (73)	p > 0.05
Poor outcome at 12-month (modified Rankin 4 - 6)	138 (51)	62 (49)	76 (53)	p > 0.05

VAI - ventriculitis-associated infection. The results are expressed as n (%).

### Outcomes

Forty-six VAI patients (36%) died during their hospital stay. Ninety-five patients with VAI (74%) presented poor outcomes at hospital discharge (poor outcome defined as mRs 4 - 6). According to the univariate analysis, the VAI was not associated with increased mortality or poor outcomes at hospital discharge (mRs 4 - 6) (Table 2).

Even in the EVD subgroup of good-grade patients (140 patients), neither mortality nor poor outcomes at hospital discharge were higher in VAI patients (60% and 52%; p > 0,05 and 18% and 25%; p > 0,05, respectively). Among good-grade patients (WFNS I - III), the incidence of VAI was 57%, and among poor-grade patients (WFNS IV - V), it was 42%.

Multivariable logistic regression analysis, with outcomes at hospital discharge, revealed that death or functional dependence (mRS of 4 to 6) was not associated with VAI (odds ratio [OR] 1.5; 95% confidence interval [95%CI] 0.7 - 2.97) after adjusting for age, poor grade, mFS 34, global cerebral edema, and pneumonia (Table 3). Similarly, unfavorable outcomes at 12 months were not associated with the VAI (OR 0.91; 95%CI 0.42 - 1.96) after adjusting for age, poor clinical grade, or aneurysm occlusion. Since long-term assessment was possible in 196 patients, we provided a table with baseline characteristics of patients with missing outcome data (Tables 2S to 5S - Supplementary Material).

**Table 3:** Uni and multivariable analysis at hospital discharge

Characteristic	Favorable n = 70	Unfavorable n = 200	p value	OR (95%CI)	p value
Age	54 (42 - 59)	56 (47 - 65)	0.015	1.02 (0.99 -1.1)	0.26
Sex female	45 (64)	153 (76)	0.047	0.55 (0.2 - 1.3)	0.16
Poor grade	10 (14)	119 (60)	< 0.001	4.97 (2.2 - 11.3)	< 0.001
Modified Fisher 3 or 4	42 (62)	162 (82)	< 0.001	2.4 (1.1 - 5.4)	0.03
Loss of consciousness	22 (31)	76 (38)	0.3		
Seizures	7 (10)	28 (14)	0.4		
Rebleed	4 (5.7)	24 (12)	0.14		
Aneurysm treatment			< 0.001		
Surgical clipping	59 (84)	98 (50)		1 (reference)	-
Endovascular coiling	7 (10)	53 (27)		2.8 (0.97 - 7.8)	0.06
No occlusion	4 (5.7)	45 (23)		4.7 (1.4 - 16.4)	0.02
Intracranial pressure monitoring	19 (27)	120 (60)	< 0.001		
Global cerebral edema	6 (8.6)	45 (22)	0.010	5.4 (1.8 - 16.3)	0.003
Cerebral infarct	15 (21)	73 (36)	0.021		
Pneumonia	8 (11)	80 (40)	< 0.001	5.1 (2 - 12.9)	< 0.001
Postoperative neurological deterioration	13 (19)	61 (31)	0.053		
Ventriculostomy associated infection	32 (46)	95 (48)	0.8	1.5 (0.7 - 2.97)	0.3

OR - odds ratio; 95%CI - 95% confidence interval. The results are expressed as the means (interquartile ranges) and n (%).

### Cerebrospinal fluid: bacteriological and biochemical profile

A total of 24 pathogens were isolated (19% of all cerebrospinal fluid [CSF] cultures), none of which were gram-positive cocci. Among the gram-negative microbes, *Acinetobacter baumannii* was predominant at 30%, followed by *Klebsiella pneumoniae* (21%), *Pseudomonas* sp., and *Enterobacter* sp. (12%).

Moreover, CSF samples from 261 patients (95%) were analyzed. The median CSF glucose, protein, lactate and WBC counts in the VAI group were 67mg/dL (IQR 47 - 82), 165mg/dL (IQR 57 - 157), 11mmol/L (IQR 3 - 8) and 1660 cells/mcL (IQR 88 - 951), respectively. In the non-VAI group, the results were 82mg/dL (IQR 65 - 96), 123mg/dL (IQR 34 - 147), 4.3mmol/L (IQR 2 - 5), and 296 cells/mcL (IQR 24 - 177) (Table 3). All CSF biochemical profile parameters were significantly different between the VAI and non-VAI groups (Table 4).

**Table 4:** Cerebral spinal fluid profile in patients with and without ventriculitis-associated infection

	VAI	Non-VAI	p value
Glucose (mg/dL)	67 (47 - 82)	82 (65 - 96)	p < 0.05
Protein (mg/dL)	165 (57 - 157)	123 (34 -147)	p < 0.05
Lactate (mmol/L)	11 (3 - 8)	4,3 (2 - 5)	p < 0.05
WBC count (cells/mcL)	1.660 (88 - 951)	296 (24 - 177)	p < 0.05

VAI - ventriculitis-associated infection; WBC - white blood cell count. The results are expressed as the median (interquartile range).

## DISCUSSION

Our study of a large cohort from two high case-volume centers in Brazil revealed a high incidence of VAI among SAH patients who required EVDs—47%. Surprisingly, we also found no impact of the VAI on short-term mortality or long-term functional outcomes. These results highlight the current limitations for effective preventive measures and accurate diagnosis of VAI in the ICU, especially in resource-limited settings.

Aneurysmal SAH is a severe cerebrovascular event that may lead to life-long disability. The mortality rate can reach as high as 15% before hospital admission and can reach 40 - 45% thirty days after bleeding.^1,14^ After the initial insult, several complications leading to secondary brain injury can further impact outcomes, and infection is among the most common complications.^2^ Most studies have shown that the major independent and modifiable risk factor for CSF infection is the duration of EVD. Beyond 5 days, there is an increased risk of VAI compared with less than 5 days.^3,5,15-17^

Our study revealed higher VAI incidence rates (47%) than those reported in the literature (2.6% - 36%).^15,18-24^ This difference is probably related to the nonstandardization and limited availability of diagnostic resources available in low- and middle-income countries in comparison to high-income countries. Unlike our initial hypothesis, our study revealed no impact on mortality or functional outcomes at discharge or at one year. Even in the subgroup with a better clinical grade at disease presentation, there were no significant differences in mortality or outcome. Most studies are conflicting regarding the associations of the VAI with increased mortality and worse neurological outcomes. While some studies reported a significant increase in mortality, costs, and length of stay,^3,6,25^ others reported no difference in mortality, only an impact on cost and length of hospital stay.^8,16^ In our study, VAI diagnosis did require microbiological criteria, which may have led to overdiagnosis and potentially overtreatment; this may also explain the high mortality rate and the lack of association with outcomes. Finally, the severity of the initial bleeding, measured by clinical and imaging scores, remains the most important determinant of outcomes after SAH.

In contrast with studies in which gram-positive Staphylococci were the most common VAI etiology,^4,26^ we isolated mostly gram-negative Bacillus. This pattern has been observed in other sources of nosocomial infections from low- and middle-income countries.^8,27^ In particular, *Acinetobacter* spp. are commonly isolated from nosocomial infections in Brazil.^28^

Our study has several limitations. First, both institutions involved are reference centers, and most patients are admitted 24 hours after ictus. The delay in transfer may introduce selection bias, as patients with a very severe disease presentation, such as evidence of intracranial hypertension, may die before reaching our ICU. Second, one-year follow-up data were acquired for only 202 VAI patients (26% lost to follow-up) (Table 2). Third, only 19% of patients diagnosed with VAI had pathogens isolated from the CSF. In addition, other components of the VAI diagnostic criteria, such as fever, neurologic injury itself, and inflammatory changes in CSF findings, may also be present without infection and simulate VAI.

Several potential opportunities arise from a better understanding of the impact of the VAI in patients with SAH. Future studies should aim at novel and more accurate methods to improve VAI diagnosis, such as early molecular detection of bacteria in the blood or CSF or monitoring of blood biomarkers (e.g., procalcitonin and C-reactive protein); these new methods can improve antimicrobial stewardship and reduce multidrug resistance outbreaks.^26,29^ In addition, strategies to prevent VAI, such as the use of an EVD bundle and antimicrobial-impregnated CSF drains, can decrease costs and eventually benefit poor-grade SAH patients.^16^

## CONCLUSION

Ventriculostomy-associated infections are common infectious complications in aneurysmal subarachnoid hemorrhage patients. This study did not reveal independent associations between ventriculostomy-associated infections and mortality or functional outcomes. Nevertheless, understanding the risks of ventriculostomy-associated infections, improving diagnostic accuracy, and applying preventive measures may affect the incidence, impact on treatment and costs of this serious nosocomial infection in aneurysmal subarachnoid hemorrhage patients.
